# Modulating CAR-T cell exhaustion and fitness in acute myeloid leukemia: mechanistic metabolic and microenvironmental strategies

**DOI:** 10.3389/fimmu.2026.1863485

**Published:** 2026-06-22

**Authors:** Erden Atilla

**Affiliations:** 1 Division of Transplantation and Cellular Therapy, Sylvester Comprehensive Cancer Center, Department of Medicine, Miller School of Medicine, University of Miami, Miami, FL, United States; 2 GENYO Centre for Genomics and Oncological Research, Genomic Medicine Department, Health Sciences Technology Park, Pfizer/University of Granada/Andalusian Regional Government, Granada, Spain

**Keywords:** AML, CAR T-cell engineering, CAR T-cell exhaustion, epigenetic regulation, metabolic fitness, microenvironment, mitochondrial function

## Abstract

Chimeric antigen receptor (CAR) T-cell therapy has achieved transformative outcomes in B-cell malignancies but remains limited in acute myeloid leukemia (AML), where antigenic heterogeneity, a suppressive myeloid-driven microenvironment, and a metabolically restrictive bone marrow niche collectively impair efficacy. In AML, CAR T-cell failure is driven not only by antigen escape but by a coordinated program of exhaustion encompassing transcriptional, epigenetic, metabolic, and functional dysfunction. The AML bone marrow niche further enforces dysfunction through suppressive immune populations, inhibitory cytokines, checkpoint signaling, metabolic competition, and impaired trafficking. This review outlines a “fitness-first” framework consolidating intrinsic and extrinsic determinants of CAR T-cell performance. Optimized manufacturing, cellular programming, and microenvironmental modulation provide a unified strategy to enhance CAR T-cell fitness and enable durable therapeutic responses in AML.

## 1 Introduction

Early clinical and translational successes in B cell malignancies established chimeric antigen receptor (CAR) T as a transformative modality, but those paradigms do not map directly onto acute myeloid leukemia (AML). However, unlike CD19 in B-cell malignancies, AML exposes fundamental limits of current chimeric antigen (CAR) T cell strategies by combining antigenic heterogeneity, pervasive myeloid-driven suppression, and a marrow microenvironment that amplifies inflammatory stress (1–3). Several AML-associated antigens including CD33, CD123, CLL-1 (CLEC12A/CD371), and FLT3 have been explored in CAR T-cell therapy. CD33 is broadly expressed on AML blasts and has long been therapeutically validated by antibody-drug conjugate approaches; however, its expression on normal myeloid progenitors creates a major risk of prolonged myelosuppression and delayed hematopoietic recovery (4, 5). CD123, the interleukin-3 receptor α chain, is enriched on AML blasts and leukemic stem/progenitor populations, and CD123 CAR T cells have demonstrated potent anti-AML activity in preclinical and early clinical studies; nevertheless, CD123 expression on normal hematopoietic progenitors and endothelial compartments raises concerns for on-target/off-tumor toxicity (6–8). CLL-1, also known as CLEC12A or CD371, has emerged as one of the most attractive AML targets because it is expressed on AML blasts and leukemic stem cells while being largely absent from normal hematopoietic stem cells (9, 10). Preclinical and early clinical studies of CLL-1/CD371 CAR T cells, demonstrated encouraging anti-leukemic activity, although antigen heterogeneity and variable expression across AML subclones remain important barriers to durable responses (11). FLT3 is another biologically relevant AML target, particularly in FLT3-mutated AML, where constitutive FLT3 signaling contributes to leukemogenesis and disease aggressiveness. FLT3-directed CAR T cells have shown potent preclinical efficacy with emerging early-phase clinical evaluation; however, FLT3 expression heterogeneity, antigen modulation, and potential hematopoietic toxicity remain key translational challenges (12, 13). Additional next-generation AML engineering approaches including logic-gated CARs, modular CAR platforms, SynNotch systems, and alternative immune effector platforms such as CAR-NK and macrophage-based therapies are also under investigation to improve specificity and safety (1).

Single-cell and clonal studies in AML reveal extensive intra-tumoral heterogeneity and complex tumor–stroma interactions that create uneven antigen landscapes and localized suppressive niches. There is empirical evidence that AML’s cellular architecture favors antigen escape and localized immune suppression, arguing for multi-antigen and resilience-focused designs rather than single-antigen approaches alone (14–16). The intra-tumoral heterogeneity in AML accelerates exhaustion when a CAR-T population is repeatedly driven by high-density clones while low-density clones persist. Exhaustion is not a single marker but a coordinated program that spans surface phenotype, transcriptional and epigenetic state, metabolic competence, and functional performance. It was demonstrated that impaired mitochondrial fitness and limited metabolic flexibility accelerate exhaustion under chronic stimulation, a dynamic that is particularly relevant in the nutrient-restricted, hypoxic marrow of AML (17–19).

A major practical limitation in AML CAR T-cell therapy is the poor quality of autologous T cells obtained from heavily pretreated patients, who frequently present with profound lymphopenia, chronic antigenic stimulation, systemic inflammation, immunosenescence, and baseline T-cell dysfunction resulting from prior chemotherapy, hypomethylating agents, stem cell transplantation, and the leukemic microenvironment itself (20). Multiple studies have demonstrated that AML patient-derived T cells exhibit impaired proliferative capacity, reduced cytokine production, mitochondrial dysfunction, altered metabolic fitness, and increased expression of exhaustion-associated markers including PD-1, TIM-3, and TIGIT (21, 22). These abnormalities may impair manufacturing efficiency, reduce the abundance of stem-like and memory-associated subsets, and predispose CAR T-cell products toward early terminal differentiation and exhaustion. In addition, prolonged ex vivo expansion of dysfunctional autologous T cells may further exacerbate metabolic stress and compromise *in vivo* persistence. These challenges have increased interest in allogeneic or “off-the-shelf” CAR platforms generated from healthy donor-derived T cells, which may provide superior baseline fitness, improved manufacturing consistency, and enhanced scalability. However, allogeneic strategies introduce additional challenges including graft-versus-host disease, host-versus-graft rejection, and the need for complex gene-editing approaches to minimize alloreactivity (23, 24). Collectively, these findings further support the concept that baseline T-cell quality is a critical determinant of CAR T-cell fitness and therapeutic durability in AML.

Taken together, these lines of evidence shift the central question for AML from “Which antigen?” to “How do we engineer CAR-T cells that retain functional capacity under simultaneous antigenic, metabolic, and inflammatory stress?” The answer requires integrating manufacturing choices that preserve stem-like subsets (25–27), metabolic and transcriptional interventions that sustain mitochondrial and epigenetic plasticity (17, 28), and microenvironmental modulation that reduces suppressive signaling and trafficking barriers (29, 30). Regulatory and translational pathways must also adapt: AML programs will need robust preclinical safety packages and contingency controls for potent armoring strategies (31). This review summarizes the mechanistic, metabolic and microenvironmental strategies that modulate CAR-T cell exhaustion and fitness in AML with the goal of mapping measurable assays to prioritized engineering interventions and translational pathways. The “fitness-first” framework proposed here organizes AML CAR-T optimization into three interconnected domains: (A) intrinsic cellular programming established during manufacturing and engineering, (B) metabolic and epigenetic resilience mechanisms that preserve functional plasticity, and (C) extrinsic modulation of the AML bone marrow microenvironment to reduce suppressive pressure. This narrative review was based on literature searches performed in PubMed and Google Scholar using combinations of keywords including AML, CAR T-cell exhaustion, metabolic fitness, microenvironment, mitochondrial function, epigenetic regulation, and CAR T-cell engineering.

## 2 AML blasts and microenvironment as a driver of CAR-T failure

New era of high-resolution cellular profiling reinforces the need for “fitness-first” CAR-T engineering which refers ability to maintain persistence, proliferative capacity, metabolic function, serial killing activity, and resistance to exhaustion. Initially, T cell exhaustion has been+ defined as a coordinated program of inhibitory-receptor expression, altered transcriptional networks, and reduced proliferative and effector capacity. Marker panels alone cannot distinguish progenitor exhausted cells from terminally exhausted cells that are epigenetically fixed; however, they provide important entry points for deeper interrogation (32–34). Co-expression of PD-1, TIM-3, LAG-3, TIGIT and ectonucleotides such as CD39 identifies T cells exposed to chronic stimulation. Progenitor like exhausted cells (Tpex) retain memory and stemness-associated genes, including TCF7, SELL, IL7R, CCR7, MYB, SLAMF6, and CD27 expression and a transcriptional program permissive of reinvigoration, whereas terminally exhausted cells express TOX, NR4A family members, and AP-1/BATF-associated programs that correlate with limited plasticity. Chen et al. (35), Alfei et al. (36), and Beltra et al. (37) map these relationships and show how subset composition predicts response to checkpoint modulation; products with a measurable TCF1^+^ progenitor pool are more likely to regain function under stress.

The AML bone-marrow niche is an active, multi-modal regulator of CAR-T failure, rather than a passive microenvironment. Three convergent features—metabolic stress, suppressive cellular networks, and soluble inhibitory mediators—accelerate transcriptional and functional collapse of adoptively transferred T cells (38, 39). Single-cell atlases of human AML reveal a spectrum of malignant states and stromal interactions that vary between patients and within clones, including differentiated AML cells that express immunomodulatory programs and directly suppress T cells. This was elegantly demonstrated by van Galen et al. when the T cells activation was reduced up to 5-fold in a dose dependent manner in the presence of AML cell lines as well as CD14+ cells from AMLs strongly inhibited T-cell activation *in vitro* (14). The well-defined expression of checkpoints-PD-L1 and CTLA-4 on tumor cells interacts with receptors on T cells that inhibit the T cell activation although checkpoint expressions were detected lower in AML compared to solid tumors (40). Upregulation of TIM3, LAG3 and TIGIT on AML blast and leukemic stem cells contribute to inhibition of T-cell activation and proliferation (41–43). Histone acetylation at immune-regulatory loci upregulates PD-L1 expression in FLT3-ITD AML (44). RUNX1 mutant and AML with KMT2A rearrangement promote exhausted T-cell infiltrates (44–47). AML cells frequently activate the PI3K/AKT/mTOR axis, which inhibits T cell effector function (48). Aberrant B-catenin activation has been shown to suppress dendritic cell maturation, reducing antigen presentation and impairing T-cell priming (45, 49). Moreover, studies have shown that myeloid leukemia cells induce downregulation of CAR molecules through leukemia derived Galectin-1 (50). Based on these findings, strategies such as inhibiting lysosomal degradation or actin polymerization effectively alleviate CAR downregulation and restore T cell function.

AML reshapes marrow immune composition toward suppressive myeloid and regulatory populations that blunt CAR-T expansion and promote exhaustion. In AML, the number of Tregs was shown to be increased and macrophages polarized toward M2- like phenotype (14, 51). Multiple studies document recruitment and functional reprogramming of MDSCs, Tregs, and leukemia-associated macrophages that secrete inhibitory mediators and consume key nutrients, creating localized pockets of suppression that limit CAR-T infiltration and function (52). Mechanistically, these cells act through contact-dependent inhibition, metabolic competition (e.g., arginase/IDO pathways), and cytokine-mediated signaling (TGF-β, IL-10) that together accelerate the transition from progenitor-like to terminally exhausted transcriptional states. Fitness requires not only initial expansion but also sustained presence in the marrow microenvironment under nutrient and suppressive pressure. Single-cell AML studies show how niche heterogeneity shapes persistence requirements (14–16).

At metabolic regulation level, AML leukemic cells competitively uptake key nutrients from microenvironment leading to functional impairment and proliferative damage to CAR-T cells due to nutrient deprivation (53). This is characterized by downregulation of key molecules including GLUT1 and mitochondrial TFAM, thereby inhibiting mitochondrial biogenesis. Furthermore, the marrow niche imposes chronic energetic stress that limits T-cell oxidative capacity and spare respiratory reserve. Scharping et al. showed that tumor-exposed T cells show progressive loss of mitochondrial mass and function driven by niche signals that repress PGC-1α and mitochondrial biogenesis; reprogramming PGC-1α restores intratumoral metabolic and effector competence (17). Early metabolic skewing is an upstream event in exhaustion: developing exhausted T cells display restricted glucose uptake, depressed mitochondrial respiration, and PD-1–linked repression of PGC-1α, establishing bioenergetic insufficiency before overt functional collapse (18). Spare respiratory capacity, mitochondrial mass and membrane potential, and the ability to upregulate oxidative phosphorylation under stress are central to sustained function. ROS handling, fatty-acid oxidation capacity, and the ability to switch between glycolysis and OXPHOS under nutrient restriction determine whether a CAR-T cell can operate in hypoxic, low-glucose marrow niches. Ma et al. (28) and Jones et al. (40) provide frameworks for metabolic profiling relevant to AML. Unlike many solid tumors that rely on glycolysis, acute myeloid leukemia (AML) cells depend on oxidative phosphorylation and glutaminolysis for energy production, with glutaminase playing a key metabolic role (54).

**Figure 1:**
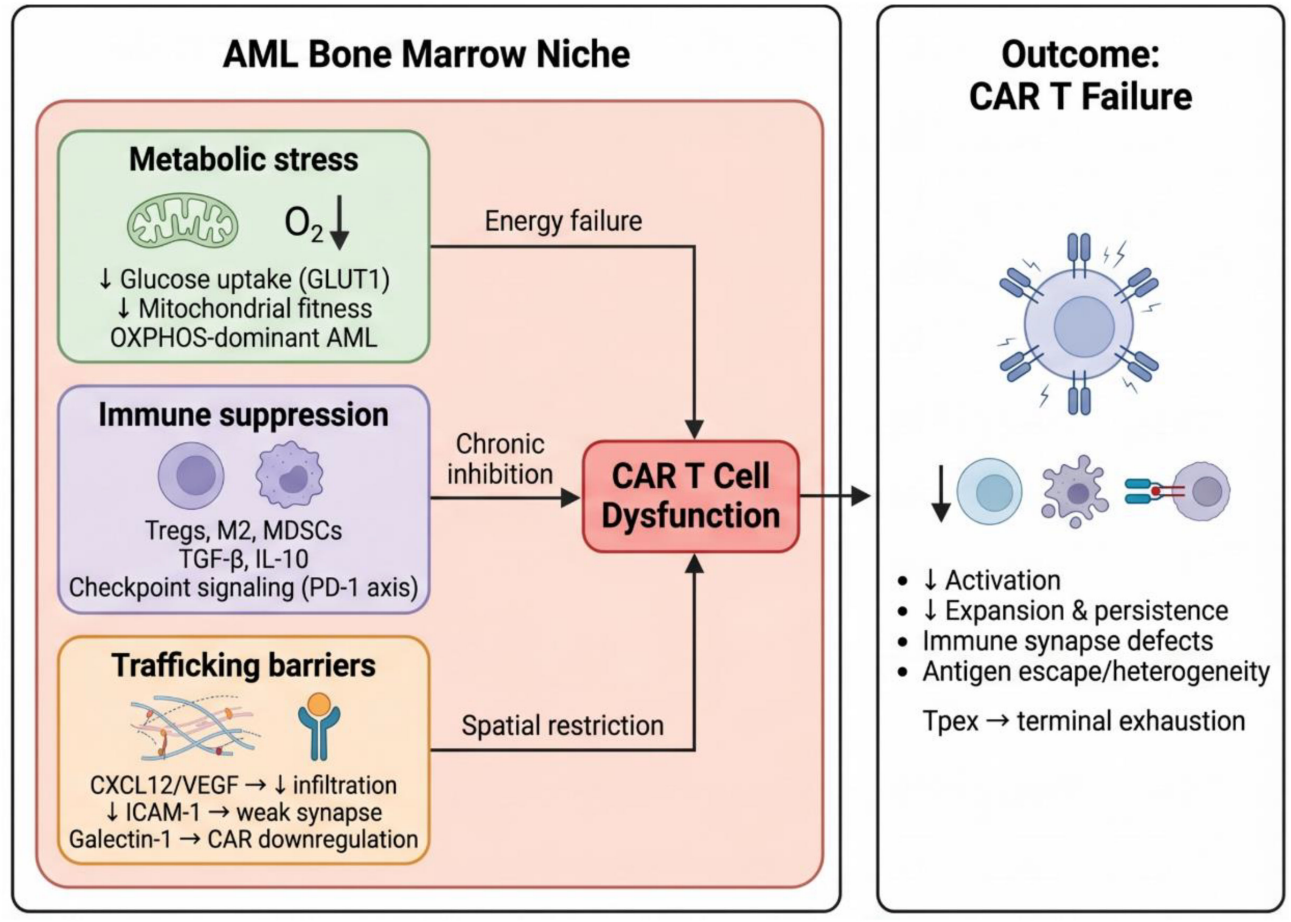
AML microenvironment integrates metabolic stress, immune suppression, and trafficking barriers to drive CAR T cell exhaustion and therapeutic failure (Created by Biorender).

Post-translational modification of chemokines and altered chemokine gradients limit effective CAR-T trafficking into marrow niches. Alterations in adhesion and signaling molecules play a critical role. Extrapolated from solid tumor models, mesenchymal stromal cells (MSCs) and fibroblasts in the bone marrow release cytokines such as CXCL12 and VEGF that suppress T cell infiltration (55). A study revealed that CD54 (ICAM-1) surface marker on resistant AML was significantly reduced leading to less contact between LFA-1 receptor on CD4 T cells and CD54 on AML cells (56). Dipeptidyl peptidase-4 (DPP4) -mediated truncation of CXCL10 reduces CXCR3-dependent lymphocyte recruitment in solid tumor preclinical models (29). Practically, trafficking deficits produce spatially heterogeneous antigen exposure: CAR-T cells that reach high-antigen pockets undergo intense chronic stimulation while other tumor clones persist in poorly infiltrated niches, a pattern that accelerates exhaustion through uneven cumulative signaling (**Figure 1**).

## 3 T-cell intrinsic programming during CAR-T manufacturing

Manufacturing choices set the baseline intrinsic program that determines whether a CAR-T product will resist exhaustion in the AML marrow niche. Preservation of less-differentiated, stem-like T cells during selection, activation, and expansion correlates with superior *in vivo* expansion, serial cytotoxicity, and metabolic reserve; these relationships are reproducible across clinical and preclinical studies and should be the primary objective of AML-directed manufacturing. Naïve and central memory (T_CM_) subsets, and T memory stem–like (T_SCM_/TCF1^+^) cells, provide proliferative reserve and multipotency that delay terminal differentiation under chronic stimulation (25). Enrichment for CD45RA^-^/CCR7^+^ or CD45RA^+^/CD62L^+^ T_SCM_ phenotypes by selective sorting or by using minimally activating stimulation conditions that avoid rapid effector differentiation (57). Release criteria may include the fraction of TCF1^+^ cells and the ratio of T_SCM_/T_CM_ to effector-phenotype cells (26). Demethylating agents (such as decitabine and azacitidine) induce T cell differentiation toward activated and memory phenotypes (58).

Strong, sustained TCR/CD3 and costimulatory signaling drives rapid effector differentiation and metabolic exhaustion; moderated activation (reduced anti-CD3/CD28 bead density or shorter stimulation windows) preserves stem-like features (27). IL-7 and IL-15 favor maintenance of T_SCM_/T_CM_ phenotypes and mitochondrial fitness compared with high-dose IL-2, which promotes terminal differentiation and glycolytic bias (26, 27). We previously demonstrated that cytokine armoring (e.g., IL-15) can preserve less-differentiated states and metabolic fitness (59). Early clinical data suggests that CLL-1 CAR T cells secreting IL18 improve *in vivo* expansion and anti-tumor activity even at low doses (60). Cytokine armoring strategies may also enhance inflammatory complications including cytokine release syndrome (CRS) and immune effector cell associated hemophagocytic lymphohistiocytosis (IEC-HS) like toxicities (59, 61).

Knockout or modulation of exhaustion-associated transcription factors (NR4A family, TOX) or overexpression of resilience factors (c-Jun, BATF modulation) can markedly improve function in syngeneic models but carry risks of dysregulated activation and safety concerns (35, 62). Use of inducible or tunable expression systems, transient mRNA delivery, or epigenetic modulators rather than permanent constitutive edits when safety data are limited (37). Any permanent genomic modification must be accompanied by rigorous off-target analysis, functional safety switches, and *in vivo* toxicity studies tailored to AML’s marrow context (63).

Manufacturing for AML must prioritize preservation of stem-like subsets, transient metabolic conditioning that enhances mitochondrial reserve, and cautious, tunable transcriptional edits—each validated by a harmonized battery of phenotypic, metabolic, and serial-challenge functional assays (17, 25, 59).

## 4 Metabolic and mitochondrial interventions

AML’s marrow niche imposes bioenergetic constraints that accelerate CAR-T collapse; therefore metabolic and mitochondrial interventions are central to any “fitness-first” engineering strategy. Mitochondrial biogenesis and spare respiratory capacity (SRC) determine whether a T cell can sustain repeated effector cycles in nutrient-poor, hypoxic marrow. Tumor-exposed T cells lose mitochondrial mass and PGC-1α expression; restoring PGC-1α rescues intratumoral function. Enforced PGC-1α expression or transient pharmacologic induction increases mitochondrial mass and SRC, improving persistence and serial killing in preclinical models (17). Short-term exposure to AMPK activators, mTOR modulators, or PGC-1α inducers during expansion can shift cells toward oxidative metabolism and enhance mitochondrial mass without permanently altering differentiation programs (28). Alternatively, researchers employed lentivirus-mediated gene transfection technology to achieve overexpression of SLC2A1 and/or TFAM genes in T cells to enhance glucose uptake capacity of T cells, promoting IL-2 release and reduces cellular exhaustion (53). The glucose transporter GLUT5 restores the metabolic fitness of CAR T cells by enabling fructose metabolism, thereby supporting glycolytic reprogramming and tricarboxylic acid cycle repair in AML (64). Exposing cells to elevated extracellular K^+^ or mimicking functional caloric restriction can preserve stemness and epigenetic features that favor long-term function. K^+^-driven functional starvation preserves stem-like programs and improves adoptive transfer outcomes (19). Reducing cholesterol uptake or ER-stress signaling prevents cholesterol-driven checkpoint upregulation and exhaustion. Cholesterol in the TME induces exhaustion; lowering cholesterol restores function in chronic viral infection model (36).

Seahorse OCR/ECAR profiling and mitochondrial membrane-potential dyes with serial killing assays to confirm that metabolic shifts translate to functional resilience (28). Interventions should be transient and titrated to avoid permanent off-target effects; validate reversibility by measuring phenotype and function after a drug-free rest period (65).

## 5 Epigenetic and transcriptional tuning

Epigenetic and transcriptional programs are the molecular fulcrum that convert repeated antigenic and inflammatory signals into durable functional states. In CAR-T biology these programs determine whether cells remain plastic and reinvigoratable or become fixed in terminal exhaustion; therefore, tuning transcriptional networks and chromatin accessibility is a high-leverage strategy to preserve fitness in AML. Mapping these states in CAR-T products and in recovered cells after infusion provides a mechanistic biomarker for likely durability and for selecting appropriate interventions (37). mRNA-based, inducible, or degron-tagged expression systems permit temporal control of transcriptional modulation, offering efficacy benefits while limiting permanent perturbation (37, 65). If scATAC/scRNA profiling shows a substantial TCF1^+^ progenitor pool, transient transcriptional modulation or localized checkpoint control is likely to yield durable benefit; if terminal epigenetic signatures dominate, more aggressive chromatin remodeling or combined metabolic rescue will be required (33, 65). Permanent transcriptional edits can produce off-target immune dysregulation, autoimmunity, or altered homeostasis; epigenetic drugs lack locus specificity and can affect hematopoietic compartments. Thorough off-target profiling, inducible control elements, and safety switches are essential mitigations (65, 66).

Key transcriptional nodes implicated in exhaustion include TOX, the NR4A family, BATF/AP-1 complexes, and modulators of TCF1-driven stemness supported by broader T-cell exhaustion literature (35, 36). Interventions can be grouped into (a) reduction of pro-exhaustion drivers (NR4A/TOX modulation), (b) augmentation of stemness programs (TCF1, BCL6), and (c) epigenetic remodeling (targeting histone modifiers or chromatin readers to preserve plasticity). Each approach has distinct mechanistic effects: transcription factor edits change signaling responsiveness, whereas epigenetic modulators alter the accessibility landscape that governs long-term program stability (34, 35). c-Jun overexpression restores AP-1 balance, resists exhaustion, and enhances antitumor activity in preclinical studies; BATF tuning similarly shifts differentiation away from terminal states (60, 67). Short courses of histone-deacetylase inhibitors, BET inhibitors, or locus-specific epigenome editors can reopen closed chromatin at key loci and restore responsiveness in progenitor pools; however, systemic epigenetic drugs affect many cell types and require careful dosing and timing (33, 68). DNA (cytosine-5)-methyltransferase 3 alpha (DNMT3A) is one of the first epigenetic regulators that was shown to increase T cell exhaustion. When DNMT3A was genetically knocked-out, exhaustion-resistant CAR T cells were shown to sustain expansion and cytotoxicity in preclinical CD19 and solid tumor models (69, 70). In leukemia and prostate cancer, the deletion of histone modifier, suppressor of variegation 3–9 homolog 1 (SUV339H1) drives a stem-like CAR-T cells (71, 72). Pharmacologic inhibition of GD2 CAR signaling with dasatinib reverses exhaustion and induces phenotypic, transcriptomic and enhancer of zeste homolog 2 (EZH2)-dependent changes (73).

Transcriptional and epigenetic states are metabolically coupled: acetyl-CoA, SAM, and NAD^+^ levels influence histone acetylation and methylation, thereby affecting exhaustion trajectories (28). Combining modest transcriptional edits with metabolic support (PGC-1α induction, AMPK activation) preserves both the signal (reduced pro-exhaustion transcriptional drive) and the capacity (energetic reserve) needed for durable function (17, 63).

Checkpoint and inhibitory-receptor pathways are central mediators of CAR-T dysfunction in chronic stimulation settings and engineering these axes can materially extend functional windows as preclinical evidence suggests. Approaches range from receptor disruption to contextual modulation (tunable blockade, dominant-negative receptors, or localized checkpoint sinks). The translational challenge is balancing enhanced antitumor activity with the risk of uncontrolled activation, autoimmunity, or altered homeostasis in the marrow niche (17, 59). PD-1 signaling reduces glycolysis and mitochondrial function while enforcing transcriptional programs that favor exhaustion; disrupting PD-1 can restore effector function but may not reverse epigenetic fixation in terminally exhausted cells (33, 65). Co-inhibitory receptors beyond PD-1, TIM-3, LAG-3, TIGIT and ecto-nucleotidases (CD39/CD73) act in parallel or sequentially to blunt proliferation and cytokine production; combinatorial inhibition often yields greater functional rescue than single-axis targeting but increases complexity and safety concerns (32, 34). Checkpoint expression is integrated with TOX/NR4A/BATF networks; editing receptors without addressing upstream transcriptional drivers can produce transient gains that erode under chronic stimulation (35, 66).

## 6 Targeting the AML microenvironment to preserve CAR-T fitness

AML’s bone-marrow niche actively undermines CAR-T durability through cellular suppression, soluble inhibitory pathways, and trafficking/metabolic barriers. Interventions that reshape the niche—either transiently before infusion or in parallel with CAR-T therapy—can markedly extend functional windows by reducing local suppression, improving infiltration, and lowering the cumulative signaling load that drives exhaustion.

Anti-CD25 antibodies or PI3K inhibitors (idelalisib) can selectively deplete Tregs or targeting arginase-1 expressed by MDSCs could enhance T cell function (74). Targeting CSF1R-dependent macrophages reduces local suppression and improves CAR-T infiltration and persistence in preclinical models; CSF1R inhibition has shown efficacy in improving CAR-T density and antitumor activity in resistant settings (75). AML blasts release enzymes (notably arginase II) that deplete arginine and reprogram local metabolism, directly suppressing T-cell proliferation and skewing myeloid polarization toward suppressive phenotypes. This arginase-dependent suppression is a validated mechanism in patient samples and preclinical models (74). Inhibiting arginase or IDO restores amino-acid availability and reduces immunosuppressive metabolite accumulation, improving T-cell proliferation and function in AML models. IDO inhibitors (such as epacadostat) blocked tryptophan metabolism and restore T-cell function (76). Immune checkpoint inhibitors (e.g. anti-PD-1, anti-CTLA-4 antibodies) block immunosuppressive pathways thereby reversing tumor-mediated T-cell suppression and T-cell exhaustion (77). In addition to blocking co-inhibitory pathways, the activation of co-stimulatory receptors, including CD137, glucocorticoid-induced TNFR-related protein (GITR), OX40, and CD27 are areas of interest (78). Inhibition of glutaminase—such as with CB-839 (telaglenastat)—disrupts AML metabolism and may partially relieve nutrient competition contributing to T-cell dysfunction and exhaustion within the marrow microenviroenment (54).

Post-translational modification of chemokines reduces effective lymphocyte recruitment. Pharmacologic DPP4 inhibition preserves active CXCL10, enhances CXCR3^+^ T-cell infiltration in solid tumor models and improved outcomes when combined with adoptive T-cell transfer or checkpoint blockage (79). TGF-β and other soluble mediators in the marrow blunt effector differentiation and promote regulatory programs; converting or blocking these signals can restore function but risks systemic effects if not localized (80). Engineering CAR-T cells to convert suppressive ligands into costimulatory signals (for example, TGF-β switch receptors) or to express local cytokine sinks can rewire inhibitory cues into activation or neutralize them locally. Preclinical switch-receptor designs preserve mitochondrial fitness and effector programs in suppressive TMEs (81, 82). Targeting Rac1GTPase, a central regulator of cytoskeletal dynamics that controls membrane protrusion and migration, in a CD33 CAR T cell constitutively express Rac1V12 model was demonstrated to increase the migration of T cells, promote T cell persistence and enhance anti-tumor efficacy *in vivo* (83).

## 7 Future directions and clinical translation

**Figure 2:**
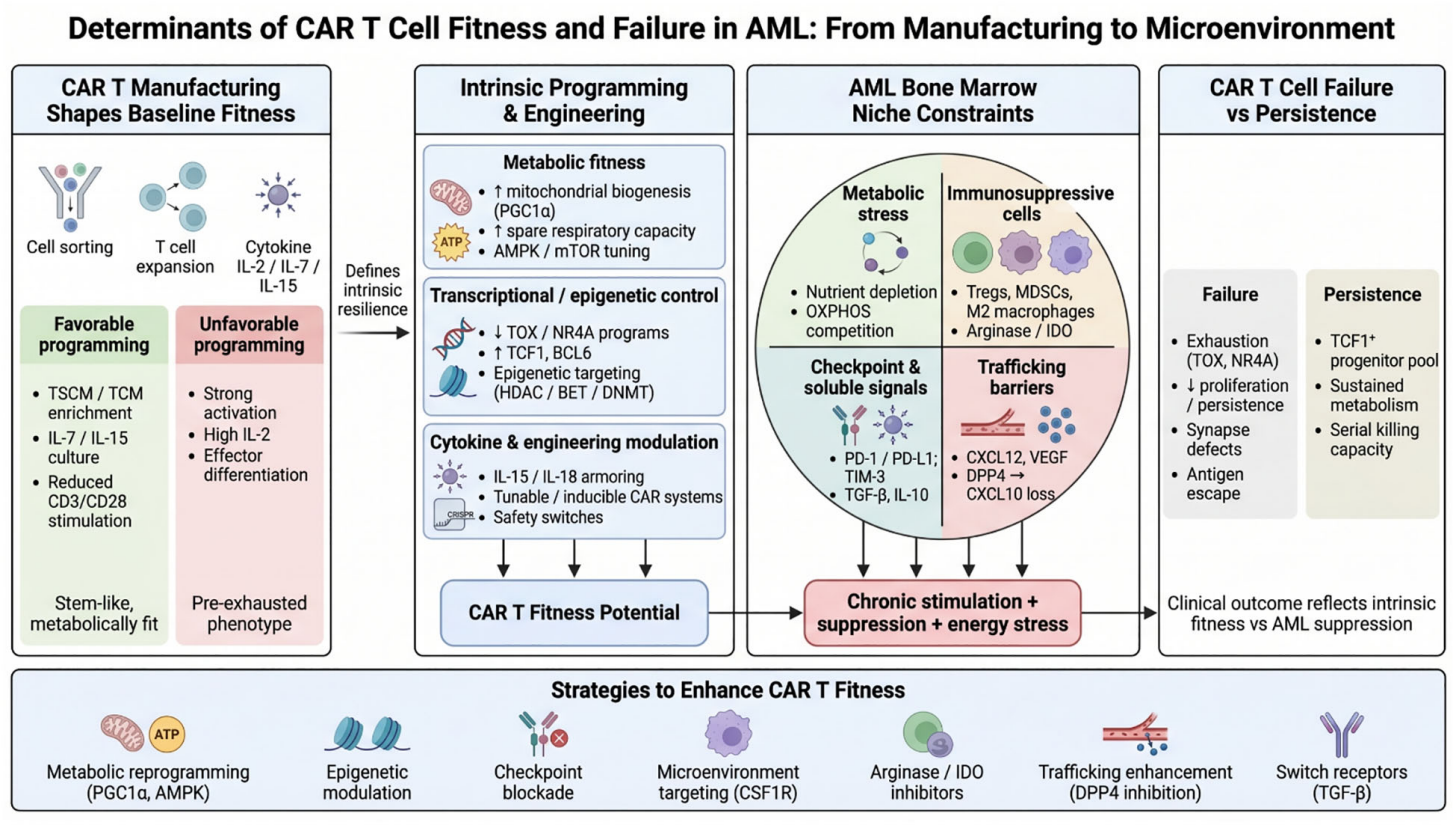
CAR T cell efficacy in AML is determined by the interplay between intrinsic programming established during manufacturing and engineering, and extrinsic pressures imposed by the AML bone marrow niche. Manufacturing strategies that preserve stem-like and metabolically fit T cells enhance resilience, whereas the AML microenvironment imposes metabolic stress, immunosuppression, checkpoint signaling, and trafficking barriers that drive exhaustion. Integrated interventions targeting both intrinsic fitness and extrinsic suppression are required to achieve durable therapeutic responses (Created by Biorender).

AML highlights the structural limitations of current CAR T-cell paradigms by combining antigenic heterogeneity, myeloid-driven immunosuppression, and a metabolically restrictive bone marrow niche, all of which accelerate T-cell exhaustion and impair long-term persistence. Consequently, durable responses in AML are likely to depend less on single-antigen targeting alone and more on engineering CAR T cells with sustained functional fitness capable of withstanding simultaneous antigenic, metabolic, and inflammatory stressors (14–16) (**Figure 2**; **Table 1**). However, enhanced fitness alone may not be sufficient to overcome the narrow therapeutic window associated with AML-directed CAR T-cell therapy. Strategies designed to increase persistence, proliferative capacity, or cytokine support may paradoxically exacerbate prolonged myelosuppression and hematopoietic toxicity when antigen selectivity is inadequate. Therefore, next-generation AML CAR T-cell development must integrate both fitness-enhancing and toxicity-mitigation strategies, including improved antigen discrimination, controllable signaling platforms, transient or regulatable CAR expression, and safety-switch mechanisms to balance durable antileukemic activity with preservation of normal hematopoiesis. First phase I trial testing c-Jun-over expressing CAR-T cells have in relapsed/refractory AML showed enhanced antitumor activity and persistence with only one of four patients experienced grade IV toxicity (84).

**Table 1:** Summary of strategies to generate ‘fit’ CAR T cells.

Category	Strategy/intervention	Mechanism	Expected benefit for CAR T-cell fitness	Potential risks/limitations	Translational readiness
T Cell Intrinsic Programming	Stem-like subset enrichment (TSCM/TCM preservation)	Enrichment of naïve and memory-like T-cell populations during manufacturing	Improved persistence, proliferative capacity, serial killing, resistance to exhaustion	Increased manufacturing complexity, cost, risk of production failure in heavily pretreated AML patients	Early clinical/translational
	Cytokine support	Promotes memory-like differentiation and mitochondrial fitness	Enhanced persistence and metabolic resilience	CRS, IEC-HS/HLH-like toxicity, excessive inflammatory activation	Preclinical to early clinical
	c-Jun overexpression	Restores AP-1 signaling balance and limits exhaustion programs	Increased persistence and resistance to exhaustion	Uncontrolled activation, prolonged immune stimulation	Early clinical
Epigenetic/Transcriptional Tuning	NR4A/TOX modulation	Reduces exhaustion-associated transcriptional programs	Enhanced cytotoxicity and functional persistence	Dysregulated activation, altered immune homeostasis	Preclinical
	PD-1/checkpoint disruption	Removes inhibitory signaling pathways	Restored effector function and metabolic activity	Autoimmunity, uncontrolled activation, inflammatory toxicity	Early clinical
	Dasatinib-mediated transient rest	Temporarily suppresses CAR signaling to reverse exhaustion	Restores functional and epigenetic plasticity	Potential temporary loss of antitumor activity	Translational/early clinical
	DNMT3A deletion	Alters epigenetic exhaustion programming	Sustained expansion and cytotoxicity	Permanent genomic editing risks, off-target effects	Preclinical
	SUV39H1 deletion	Promotes stem-like chromatin state	Improved persistence and less differentiated phenotype	Epigenetic instability, genomic safety concerns	Preclinical
Metabolic and Mitochondrial Interventions	PGC-1α induction	Restores mitochondrial biogenesis and oxidative metabolism	Improved mitochondrial reserve and persistence	Potential metabolic imbalance, limited AML-specific validation	Preclinical
	AMPK/mTOR modulation	Metabolic reprogramming toward oxidative phosphorylation	Enhanced metabolic flexibility and resistance to exhaustion	Off-target metabolic effects, excessive metabolic stress	Preclinical
	GLUT5 metabolic engineering	Enables fructose metabolism and glycolytic support	Restores metabolic fitness in nutrient-poor environments	Limited *in vivo* and AML-specific validation	Experimental/preclinical
Targeting Microenvironment	Arginase inhibition	Restores arginine availability in AML microenvironment	Improved proliferation and effector activity	Systemic metabolic effects	Preclinical
	CSF1R inhibition	Reduces suppressive macrophage populations	Enhanced CAR T-cell infiltration and persistence	Off-target immune effects	Preclinical/translational
	DPP4 inhibition	Prevents CXCL10 truncation and improves trafficking	Enhanced T-cell infiltration into tumor niches	Limited AML-specific validation	Preclinical
	TGF-β switch receptors	Converts suppressive signaling into activation signals	Preserved effector function in suppressive microenvironment	Excessive activation, off-target immune effects	Preclinical

Translating “fitness-first” CAR-T strategies for AML requires coordinated advances across combination biology, biomarker-driven trial design, and regulatory risk-management. The goal is not merely to increase short-term cytotoxicity but to shift the therapeutic axis toward durable functional competence in the marrow niche by combining modest, reversible cell-intrinsic edits with targeted, time-limited microenvironmental modulation. Rational combinations should be mechanistically complementary rather than additive in toxicity. For AML this typically means pairing (a) manufacturing and metabolic levers that preserve mitochondrial reserve and TCF1^+^/T_SCM_ fractions, (b) tunable checkpoint or transcriptional modulation to blunt early exhaustion signaling, and (c) short-course niche adjuncts (trafficking enhancers, myeloid re-programmers, or metabolic enzyme blockers) that reduce localized suppressive pressure. Such layering reduces the need for aggressive permanent edits and targets the critical engraftment window when CAR-T cells are most vulnerable. Trial designs should therefore prioritize adaptive, biomarker-guided arms that allow escalation of intrinsic edits only in patients whose early correlative data predict rapid collapse (31, 85).

Key clinical trial design elements include pre-specified fitness endpoints (not only response rate): product TCF1^+^ fraction, spare respiratory capacity, early marrow CAR-T infiltration, and ex vivo serial-challenge durability. Early on-treatment biopsies (Day +7–14) provide decision points for adaptive escalation or adjunct addition. Randomized or biomarker-enriched cohorts improve signal detection for adjuncts that primarily affect trafficking or niche composition. A multi-modal biomarker strategy links product state, niche context, and early *in vivo* trajectory. Pre-infusion product metrics should include TCF1/T_CM_ fractions and metabolic readouts (OCR/SRC, NAD^+^/ATP ratios). Integrated metabolomics and scATAC/scRNA profiling map metabolic-epigenetic coupling and identify patients likely to benefit from metabolic or epigenetic augmentation (14, 28).

It should be noted that prospective subset selection may reduce starting cell numbers, introduce additional manipulation and GMP-compatible sorting steps, and increase manufacturing complexity, cost, batch-to-batch variability, and the risk of production failure, particularly in heavily pretreated AML patients with lymphopenia or dysfunctional T-cell compartments (86, 87). Similarly, transient metabolic conditioning strategies require careful optimization of dose, timing, washout procedures, and comparability testing to avoid unintended effects on expansion kinetics, exhaustion, or product safety (88, 89). Expanded release criteria incorporating phenotypic, metabolic, and serial challenge functional assays may better capture CAR T-cell fitness than conventional single end-point cytotoxicity assays; however, these assays must remain reproducible, validated, scalable, and compatible with clinically relevant vein-to-vein timelines (87).

Regulatory strategy must reflect the risk spectrum of interventions. Favor transient, inducible, or non-genetic modalities for first-in-human testing; reserve permanent multi-locus edits for later cohorts after robust safety and off-target profiling. IND packages should include marrow-relevant toxicology, off-target editing analyses, and correlative biomarker plans that demonstrate mechanisms without compromising hematopoiesis. Safety monitoring must emphasize hematologic recovery, infection surveillance, and predefined management for amplified inflammatory toxicity (65). This “fitness-first” framework provides a coherent path toward durable AML responses by integrating manufacturing, intrinsic engineering, and microenvironmental modulation into a unified translational strategy.
